# HOTTIP Mediated Therapy Resistance in Glioma Cells Involves Regulation of EMT-Related miR-10b

**DOI:** 10.3389/fonc.2022.873561

**Published:** 2022-03-24

**Authors:** Zhang Li, Ming Li, Pengcheng Xia, Zhiming Lu

**Affiliations:** Department of Clinical Laboratory, Shandong Provincial Hospital, Cheeloo College of Medicine, Shandong University, Jinan, China

**Keywords:** glioma, HOTTIP, miR-10b, temozolomide, EMT

## Abstract

The advanced grade glioblastomas are characterized by dismal five-year survival rates and are associated with worse outcomes. Additionally, resistance to therapies is an additional burden responsible for glioma associated mortality. We studied the resistance against temozolomide (TMZ) as a surrogate to understand the mechanism of therapy resistance in glioma cancer cells. Screening of three glioma cells lines, A172, LN229 and SF268 revealed that SF268 glioma cells were particularly resistant to TMZ with the IC-50 of this cell line for TMZ ten times higher than for the other two cell lines. A role of lncRNAs in glioma progression has been identified in recent years and, therefore, we focused on lncRNAs for their role in regulating TMZ resistance in glioma cancer cells. lncRNA HOTTIP was found to be particularly elevated in SF268 cells and over-expression of HOTTIP in both A172 and LN229 remarkably increased their TMZ IC-50s, along with increased cell proliferation, migration, clonogenicity and markers of angiogenesis and metastasis. As a mechanism we observed increased expression of miRNA-10b and mesenchymal markers Zeb1/Zeb2 and reduced expression of E-cadherin in SF268 cells indicating a role of EMT in TMZ resistance. A172 and LN229 cells with overexpressed HOTTIP also had similarly induced EMT and the elevated miR-10b levels. Further, silencing of miR-10b in HOTTIP overexpressing cells as well as the SF268 cells reversed EMT with associated sensitization of all the tested cells to TMZ. Our results thus present a case for HOTTIP in native as well as acquired resistance of glioma cells against chemotherapy, with a key mechanistic role of EMT and the miR-10b. Thus, HOTTIP as well as miR-10b are critical targets for glioma therapy, and need to be tested further.

## Introduction

Glioma is a brain tumor which is fairly common and represents about one-thirds of all brain tumors. Less than a quarter of glioma patients survive for more than five years and the median survival is less than 2 years (1). It is an aggressive cancer in adults and largely considered incurable (2). The clinical management of glioma patients involves surgical resection, if possible, followed by temozolomide (TMZ) together with radiotherapy and finally adjuvant TMZ (1). This underlines the importance of TMZ in clinical management of glioma patients, particularly in view of the use of TMZ for almost two decades (3). Despite this importance of TMZ in glioma, the resistance against this drug is fairly common with almost half of the patients failing to respond to it (4). Even with the relative wealth of information on possible mechanisms that can lead to TMZ resistance, including epigenetic ones that include aberrations in DNA methyltransferase, the topic remains poorly understood (5). In view of the high mortality cause by therapy resistance, it is important to better understand the overall mechanism of TMZ resistance in gliomas.

LncRNAs are increasingly being investigated for their role in diagnosis, prognosis and treatment of cancers (6), including gliomas (7). They are also being investigated for possible role in determining resistance against therapies (8, 9), including in gliomas (8, 10). In view of this knowledge, we focused our study to understand the lncRNA mediated TMZ resistance in glioma. For the model system, we hypothesized that the glioma cells with endogenous resistance against TMZ might be the best models, relative to TMZ-sensitive glioma cells. Thus, we screened a number of cell lines in order to find the appropriate working model. Our study identified lncRNA HOTTIP as the lncRNA of interest. Previously, lncRNA HOTTIP has been shown to mediate hypoxia-induced EMT (epithelial-mesenchymal transition) in glioma cells U87 and U251 (11), thus increasing confidence in our work. We further elucidated the mechanism of TMZ resistance by looking at underlying mechanism and confirmed EMT induction that also involved microRNA-10b (miR-10b).

## Materials and Methods

### Cell Lines and Culture

A172 (ATCC: CRL-1620), LN229 (ATCC: CRL-2611) and SF268 glioma cell lines were all purchased from ATCC (Virginia, USA) and regularly screed for mycoplasma in the laboratory. All of these cell lines were cultured in DMEM culture medium with added FBS at 10% final concentration and added antibiotics penicillin and streptomycin at 1% final concentrations (Life Technologies, China). Cells were cultured in certified incubators at 37^0^C under humidified conditions and 5% CO_2_.

### TMZ and Dilutions

TMZ was purchased from Sigma Chemical Company (China). Stock of TMZ was prepared in DMSO at a concentration of 20mg/ml and further dissolved in culture medium, as needed for individual assays. TMZ treatment was done for a 3 day cycle as also described earlier by Perazzoli and co-workers (12).

#### HOTTIP Transfections

We transfected full-length HOTTIP into pcDNA3.1 vector (GenePharma, Shanghai, China) and the control plasmid without HOTTIP was used as a negative control, similar to the method described elsewhere (13). HOTTIP was transfected into the glioma cells, using Lipofectamine 3000 (Life Technologies, China), as per the instructions supplied by the company for the transfection reagent.

#### MTT Assay

For proliferation assay, we conducted MTT assays. Cells were seeded in 96-well plates at a density of 3500 cells/well. Once the indicated assays were done, 20 µL reconstituted MTT reagent (5 mg/mL) was added to all wells for 2 hours. Then the wells were emptied and filled with 100 µL DMSO. The optical density (OD) was measured at 570 nm using a Shimadzu colorimeter (Japan).

### Migration Assay

Migration assays were performed using QCM cell migration assay kits (Millipore, China), which assess the potential of cells to migrate colorimetrically, and as described in an earlier publication (14). Glioma cancer cells were seeded into the upper chamber with media that did not contain FBS. This initiated migration towards the lower chamber which was filled with complete media that contained 10% FBS. The cells that migrated were stained with crystal violet and absorbance was read using a Shimadzu colorimeter (Japan).

### ELISA Assays

Angiogenesis and metastasis potentials were assessed by quantitating VEGF and MMP-9, respectively, in the supernatants of the glioma cell cultures, using quantitative ELISA kits (Sigma, China). Exact protocol recommended by the manufacturer was followed and the absorbance at 450nm was read using a Shimadzu colorimeter (Japan).

### lncRNA and miRNA Detection

Total RNA was extracted using Trizol reagent (Life Technologies, China). lncRNAs as well as miRNAs (using Taqman primer-probes) were quantitated using commercially available reagents from ThermoFisher Scientific (USA). This included reagents needed for all lncRNAs, miRNAs and the controls.

### Anti-Pre-miRNA Transfections

Anti-miR-10b was purchased from ThermoFisher Scientific (USA) and transfected in cells using G-fectin (Genolution, South Korea). The protocol of manufacturer was used without any modification to accomplish successful transfection of miRNA.

#### Reverse Transcription-Quantitative Polymerase Chain Reaction (RT-qPCR)

Total RNA was extracted from the cells using Trizol reagent (Life technologies, China). cDNA was prepared by reverse transcription using 1 μg RNA. We sued Prime Script™ RT Master Mix (Takara Bio, Japan) for cDNA preparation. The mRNAs of EMT related genes were amplified using SYBR^®^ Premix Kits (Takara Bio, Japan) and quantitated using CFX96™ real-time machine (Bio-Rad, China). GAPDH was used as internal control for mRNA quantitation.

#### Statistical Analysis

All of the statistical evaluations were carried out using SPSS statistical software (SPSS Inc., Chicago, IL, USA). The values of p < 0.05 were considered significant. The experiments were repeated at least three time with atleast duplicate samples in each run. Student’s t-test was used for sample comparisons.

## Results

### TMZ Resistance Glioma Models

**Figure 1:**
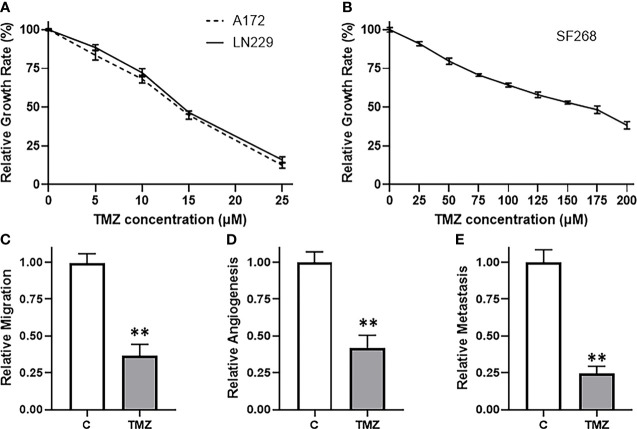
SF268 are resistant to TMZ. MTT assays were performed to test the sensitivity of **(A)** A172 and LN229 and **(B)** SF268 glioma cells against TMZ after 3 day cycle. Vehicle treated control vs. 160μM TMZ treated SF268 cells were subjected to assays for migration **(C)**, VEGF secretion **(D)** and MMP-9 secretion **(E)**. VEGF and MMP-9 were detected by ELISA as surrogates for angiogenesis and metastasis, respectively. The values of controls were regarded as ‘1’ and relative values of TMZ-treated SF268 cells are reported. C: control, TMZ: TMZ-treated SF268 cells **p < 0.01.

We started our study by looking for the most appropriate model system to investigate TMZ resistance. We screened a number of glioma cell lines and finally shortlisted three, viz. A172, LN229 and SF268 glioma cell lines. These three cell lines were selected because of their sensitivity/resistance to TMZ. MTT assays revealed that the two cell lines A172 and LN229 were relatively sensitive to TMZ (**Figure 1A**) while the third cell line SF268 was relatively resistant to TMZ (**Figure 1B**). The IC-50 values of the three cell lines were 13.7 ± 1.2 μM, 14.4 ± 1.0 μM and 155.1 ± 1.7 μM for A172, LN229 and SF268 respectively (**Table 1**). Thus, SF268 cells were quite resistant to TMZ as their IC-50 for TMZ was more than ten-times that of both A172 and LN229 cells.

**Table 1:** IC-50 values of glioma cells against Temozolomide.

Cell Line	Condition	IC-50 (μM)
SF268	Native	155.1 ± 1.7
	+i10b	100.1 ± 1.2
A172	Native	13.7 ± 1.2
	+HOTTIP	54.6 ± 1.5
	+H+i10b	26.3 ± 0.7
LN229	Native	14.4 ± 1.0
	+HOTTIP	63.8 ± 1.8
	+H+i10b	25.0 ± 0.9

+ i10b: silenced for miR-10b, +HOTTIP: transfected with HOTTIP, +H+ i10b: Transfected with HOTTIP and silenced for miR-10b.

Next, we confirmed the TMZ resistance nature of SF268 cells by carrying out a number of assays that are determinants of cancer aggressiveness. We chose a dose of TMZ that was little higher than the IC-50 of these cells i.e. 160 μM and then tested a few different parameters, viz. migration, angiogenesis and metastasis. Migration was performed using boyden chamber assay and we found that the migration was reduced by more than half in SF268 cells by the 160 μM dose (**Figure 1C**). Angiogenesis was measured by quantitating the release of biomarker VEGF by ELISA and we found reduction in release of VEGF by more than half when SF268 cells were treated with 160 μM TMZ (**Figure 1D**). Metastasis was measured by quantitating the release of MMP-9 by ELISA and our assay revealed that the used TMZ dose resulted in significantly reduced MMP-9 secretion (reduced by more than half) (**Figure 1E**). Thus, these experiments confirmed that a dose of 160 μM TMZ was more than IC-50 and significantly impacted the various parameters that lead to TMZ-resistance associated mortality.

### HOTTIP Is Elevated in TMZ Resistant Cells

**Figure 2:**
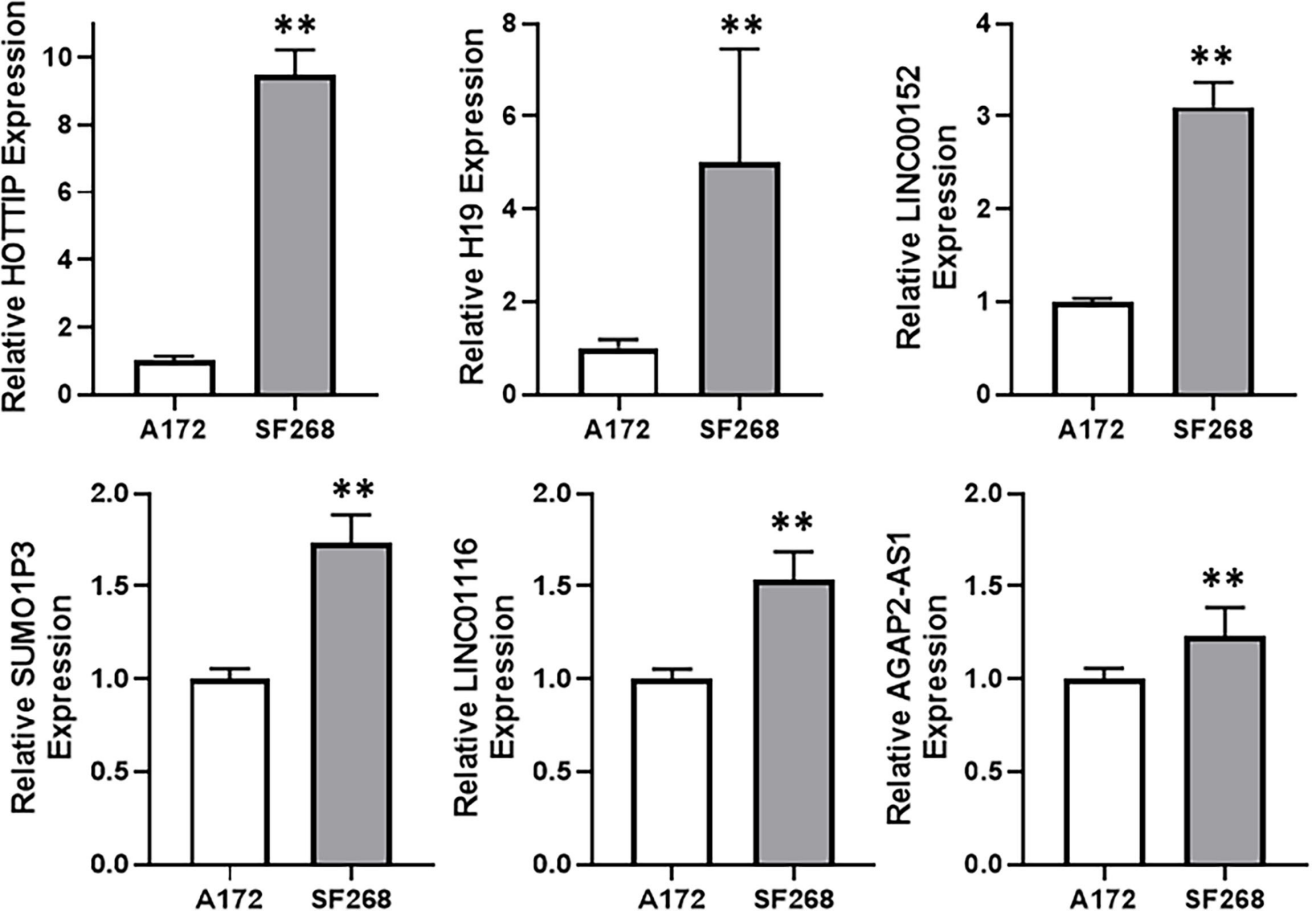
HOTTIP is elevated in TMZ resistant cells. Expression levels of different lncRNAs were evaluated in A172 vs. SF268 cells, by RT-PCR. The values in A172 cells were regarded as ‘1’ and relative values in SF268 cells are reported. **p < 0.01.

Since our aim was to find a potential lncRNA that can mediate TMZ resistance in glioma cells, we compared TMZ-sensitive A172 and TMZ-resistant SF268 cells for their expression of several lncRNAs. Our screening revealed that a number of lncRNAs, such as HOTTIP, H19, LINC00152, SUMO1P3, LINC01116 and AGAP2-AS1 were significantly overexpressed in SF268 cells, relative to A172 cells (**Figure 2**), indicating their role in mediating TMZ resistance. While AGAP2-AS1, LINC01116 and SUMO1P3 were less than doubled, lncRNAs LINC00152, H19 and HOTTIP were elevated many folds. Of these, HOTTIP was found to be particularly elevated with its levels increased more than nine-folds in TMZ-resistant SF268 cells.

### HOTTIP Induced Changes in TMZ Sensitive Cells

**Figure 3:**
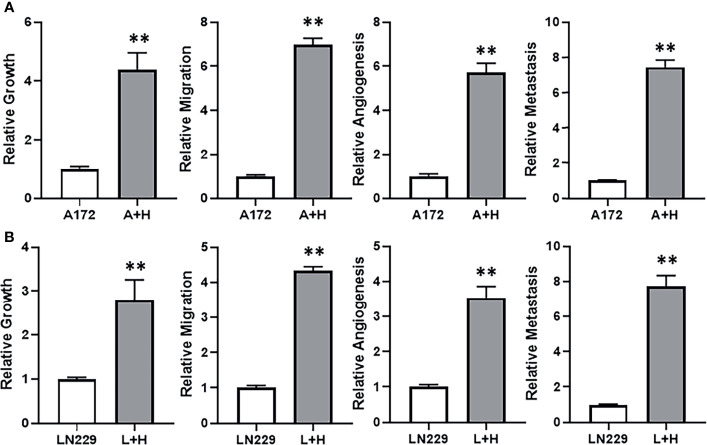
HOTTIP impacts cellular proliferation, migration and markers of angiogenesis and metastasis. A172 **(A)** and LN229 **(B)** cells were transfected with lncRNA HOTTIP and cell growth (by MTT), migration, angiogenesis (VEGF secretion by ELISA) and metastasis (MMP-9 secretion by ELISA) were measured. A+H: A172 cells with HOTTIP, L+H:LN229 cells with HOTTIP. **p < 0.01.

With the observation that lncRNA HOTTIP was relatively highly expressed in TMZ resistant SF268 cells, we hypothesized that HOTTIP was involved in determining the resistance of SF268 against TMZ. To test this hypothesis, we transfected HOTTIP in the otherwise TMZ sensitive glioma cells and tested the various cancer parameters that are connected with therapy resistance. First, we transfected A172 cells with HOTTIP and evaluated the resulting effect on cell growth/proliferation, migration, angiogenesis and metastasis. Transfection of HOTTIP into A172 cells, significantly increased their proliferation, migration as well as the secretion of VEGF and MMP-9 (**Figure 3A**). To further confirm our results, we transfected the other TMZ sensitive cells LN229 with HOTTIP as well and evaluated the same parameters. As shown in **Figure 3B**, HOTTIP transfection significantly increased the proliferation, migration and the release of VEGF and MMP-9 from LN229 cells as well. Thus, our results established a role of HOTTIP in inducing several parameters in glioma cells that can impact resistance against therapy.

### miR-10b Is Also Elevated in TMZ Resistant Cells

**Figure 4:**
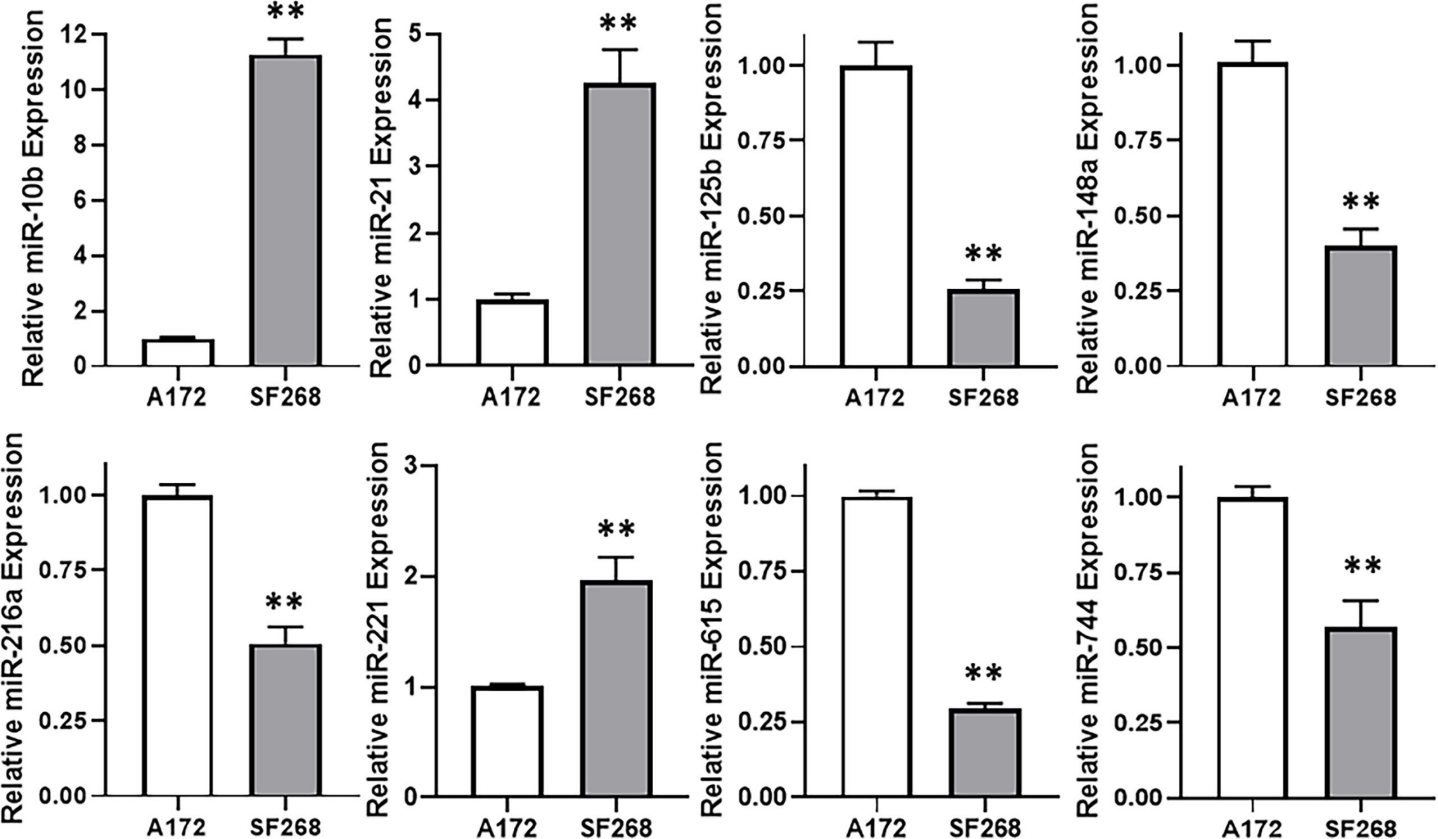
miR-10b is elevated in TMZ resistant cells. Expression levels of different miRNAs were evaluated in A172 vs. SF268 cells, by RT-PCR. The values in A172 cells were regarded as ‘1’ and relative values in SF268 cells are reported. RNU6B was used as the endogenous control miRNA. **p < 0.01.

The wealth of literature on lncRNAs in cancer has taught us that their functions involve regulation of miRNAs (15, 16). Therefore, our next task was to find a miRNA that is relevant to TMZ resistance in our glioma models. For this, we again compared TMZ-sensitive A172 and TMZ-resistant SF268 cells, this time for their expression of miRNAs. We found a number of miRNAs that were significantly different between the two cell lines and the top ones are presented in **Figure 4**. miR-10b stood out as the most overexpressed miRNA in the TMZ resistant SF268 cells with its expression more than ten-folds, compared to A172 cells. A few other miRNAs were also increased in SF268 cells which included miR-21 and miR-221. A number of other miRNAs, on the other hand, were significantly reduced in TMZ resistant SF268 cells and these included miR-125b, miR148a, miR-216a, miR-615 and miR-744. No other miRNAs was significantly changed in resistant cells as the miR-10b and, therefore, we chose this miRNA for further involvement and mechanism-based studies.

### EMT Is Induced in TMZ Resistant Cells

**Figure 5:**
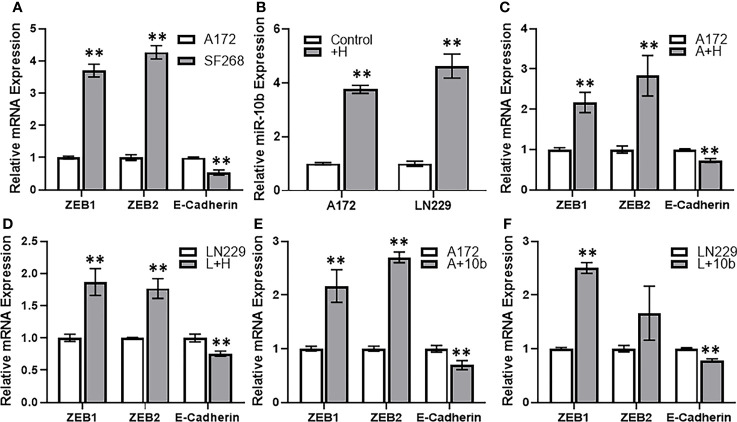
HOTTIP induces miR-10b and EMT in glioma cells. **(A)** Expression levels of EMT markers were evaluated in A172 vs. SF268 cells, by RT-PCR. The values in A172 cells were regarded as ‘1’ and relative values in SF268 cells are reported. GAPDH was evaluated as the endogenous control mRNA. **(B)** A172 and LN229 cells were transfected with lncRNA HOTTIP and expression levels of miR-10b were evaluated in control vs. HOTTIP transfected cells were evaluated by RT-PCR. The levels of miR-10b in control cells were regarded as ‘1’ and relative levels in HOTTIP transfected cells are reported. RNU6B was used as the endogenous control miRNA. Expression levels of EMT markers were evaluated in A172 cells **(C)** or LN229 cells **(D)** transfected with HOTTIP or the A172 cells **(E)** or LN229 cells **(F)** transfected with miR-10b **(D)** by RT-PCR. The values in control cells were regarded as ‘1’ and relative values in transfected cells are reported. GAPDH was evaluated as the endogenous control mRNA. +H: HOTTIP transfected cells, A+H: A172 cells with HOTTIP, A+10b: A172 cells with miR-10b. **p < 0.01.

Among many potential mechanisms that can play a part in resistance against therapy, EMT is a promising one. Therefore, we next checked for the possible involvement of EMT in the induction of TMZ resistance in glioma cells. When we compared the gene expression of various EMT markers, viz. ZEB1, ZEB2 and e-cadherin in resistant vs sensitive cells, we found that mesenchymal markers ZEB1 and ZEB2 were significantly elevated whereas the epithelial marker E-cadherin was significantly decreased in resistant SF268 cells (**Figure 5A**). We also checked if HOTTIP overexpression in sensitive cells could impact miR-10b levels, and found that overexpression of HOTTIP in both A172 and LN229 cells significantly increased miR-10b levels (**Figure 5B**). This meant that the relationship between HOTTIP and miR-10b is valid in all glioma cells that turn resistant against TMZ. In A172 cells with overexpressed HOTTIP, we also found evidence of EMT as evidenced by increased mesenchymal markers and decreased epithelial marker (**Figure 5C**). In LN229 cells as well, overexpression of HOTTIP led to increased mesenchymal markers and decreased epithelial marker (**Figure 5D**). Similar induction of EMT was also evident in A172 as well as LN229 cells when, instead of HOTTIP overexpression, they were subjected to miR-10b overexpression (**Figures 5E, F**). Again, mesenchymal markers ZEB1 and ZEB2 were increased while the epithelial marker E-cadherin was decreased. In summary, these observations increased our confidence in the finding that HOTTIP increases miR-10b and over expression of both leads to EMT in glioma cells.

### Role of miR-10b in HOTTIP Mediated TMZ Resistance

**Figure 6:**
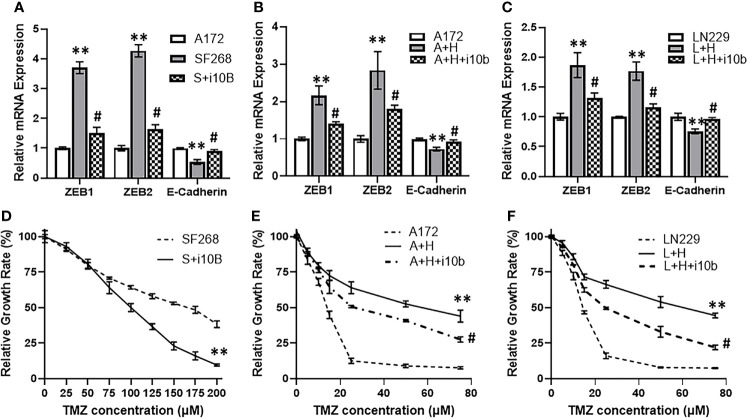
Silencing of miR-10b reverses HOTTIP effects. **(A)** Expression levels of EMT markers were evaluated in A172 vs. SF268 cells (with and without miR-10b inhibition), by RT-PCR. The values in A172 cells were regarded as ‘1’ and relative values in SF268 cells are reported. GAPDH was evaluated as the endogenous control mRNA. **(B, C)** Expression levels of EMT markers were also evaluated in A172 and LN229 cells transfected with HOTTIP (with and without miR-10b inhibition), by RT-PCR. The values in control cells were regarded as ‘1’ and relative values in transfected cells are reported. GAPDH was evaluated as the endogenous control mRNA. MTT assays were performed to test the sensitivity of **(D)** SF268, **(E)** A172 and **(F)** LN229 glioma cells against TMZ after 3 day cycle, under different conditions. S+i10b: SF268 + anti-miR-10b, A+H: A172 cells with HOTTIP, A+H+i10b: A172 cells transfected with HOTTIP and anti-miR-10b. **p < 0.01 vs control, ^#^p < 0.01 in miR-10b silenced cells vs. unsilenced (transfected with control anti-miRNA oligos) cells.

We also further experimentally confirmed our hypothesis for the involvement of miR-10b in HOTTIP mediated TMZ resistance of glioma cells. Firstly, in the comparison between resistant SF268 and sensitive A172 cells where EMT markers were found elevated in SF268 cells, we added an additional group i.e. SF268 cells with silenced miR-10b. We checked for the efficiency of miR-10b silencing and found reduction in miR-10b levels of anywhere between 62% and 45% of the non-specific controls (results not shown). We found that antagonizing miR-10b significantly reversed EMT as evidenced by significantly reduced ZEB1 and ZEB2 while significantly increased E-cadherin (**Figure 6A**). Similar observations were made when A172 cells and the LN229 cells with overexpressed HOTTIP were subjected to silencing of miR-10b. Again, EMT was reversed by silencing of miR-10b (**Figures 6B, C**). As a final experiment to firmly link miR-10b with HOTTIP mediated TMZ resistance, we compared SF268 and miR-10b silenced SF268 cells for their sensitivity to TMZ by exposing the cells to increasing concentrations of TMZ. We found that silencing of miR-10b significantly reduced the resistance of SF268 cells against TMZ (**Figure 6D**) with IC-50 value dropping to 100.1 ± 1.2 μM (**Table 1**). On similar lines, when A172 or the LN229 cells with HOTTIP overexpression were silenced for miR-10b, their IC-50 also significantly reduced (**Figures 6E, F**) dropping from 54.6 ± 1.5 and 63.8 ± 1.8 μM, respectively for the A172 and LN229 HOTTIP overexpressed cells to 26.3 ± 0.7 and 25.0 ± 0.9 μM, respectively, for the A172 and LN229 HOTTIP overexpressed cells with silenced miR-10b.

## Discussion

Glioma is an aggressive cancer with poor prognosis and outcomes, thus making it important to find novel targets of therapy. In particular, resistance against therapy, both inherent as well as acquired, such as resistance against TMZ is a major clinical challenge making the condition of patients worse and increasing the mortality. LncRNAs are quickly emerging as the molecules of interest, particularly according to reports in recent years (17, 18). This prompted us to investigate TMZ resistance of glioma as regulated by lncRNAs.

The IC-50 values of glioma cell lines that we tested and reported in this manuscript have been reported by other researchers as well. In a report published by Perazzoli and co-workers, A172 cells had an IC-50 of 14.1 μM, LN229 cells had an IC-50 of 14.5 μM and SF268 had an IC-50 of 147.2 μM (12). Our results, as reported here are in general agreement with those previously reported values as we also report A172 and LN229 cells as the cell lines sensitive to TMZ with IC-50 values with IC-50 values 13.7 μM and 14.4 μM respectively, which are very close to what was reported by Perazzoli et al. (12). Moreover, we also show that SF268 cells are comparatively resistant to TMZ with IC-50 value of 155.1 μM. Interestingly, our analysis reveal a little more than ten-times higher IC-50 value for SF268 cells, compared to A172 and LN229 cells, which is also in general agreement with the results from study by Perazzoli et al. (12).

For the lncRNAs that can positively impact glioma cells resistance against TMZ, in addition to playing a role in glioma cells’ proliferation, invasion and metastasis, we tested a total of thirty lncRNAs, based on the reported literature. The top six lncRNAs have been proven to exhibit multiple effects against glioma cells. H19 lncRNA has been shown to promote the proliferation, migration and invasion of glioma cells (19) through targeting of miR-200a. LINC00152 is similarly expressed at higher levels in gliomas where it increases proliferation and invasion (20). SUMO1P3 is also elevated in gliomas and associates with poor survival of glioma patients (21). Its knock down negatively affects proliferation and invasion of cells. LINC01116 is highly expressed in gliomas and promotes proliferation and invasiveness of glioma cells by targeting miR-744 (22). Finally, the last lncRNA, AGAP2-AS1, regulates proliferation and metastasis of glioma cells (23).

We show effect of HOTTIP on EMT. In glioma, there is one published report on the role of HOTTIP in EMT. This study (11) focused on hypoxia mediated EMT and found an important role of lncRNA in the process. HOTTIP was identified based on lncRNA array analysis between U87 glioma cells with and without hypoxia. Hypoxia was found to promote HOTTIP expression and metastasis, which also correlated with poor patient survival. However, a different mechanism was identified as this published study reported an involvement of miR-101 in HOTTIP action while we report an involvement of miR-10b. It is important to note that whereas miR-101 is sponged by HOTTIP as reported in the hypoxia report (11), we found elevated miR-10b in cells that also had increased HOTTIP levels. Additionally, the hypoxia report used U87 and U251 cells whereas we report our findings in three completely different cells, viz, A172, LN229 and SF268. Thus, a cell line effect can also not be ruled out. It might be important to conduct a study that uses all of these five cell lines. In addition to the one report on a connection between HOTTIP and EMT in glioma, there are a few other reports connecting HOTTIP with EMT in some other cancers. Examples include the effect of HOTTIP on EMT in breast cancer (24), gastric cancer (25), osteosarcoma (26), ovarian cancer (27). Such EMT induction by HOTTIP has been linked to cisplatin resistance in gastric cancer (28), thus further validating HOTTIP mediated EMT in resistance against therapies.

Interestingly, one of the other shown lncRNA, H19 also seems to affect EMT in glioma cells (19). The very indication that this H19 affects MET comes through the miRNA it targets as this miRNA is very well known to be involved in regulation of EMT (29), thus making this miRNA an attractive cancer biomarker (30). Similar to our findings reported here, H9 was found to regulate EMT marker ZEB1 (19) which thus appears to be important EMT gene regulated by lncRNAs. Similarly, lncRNA SUMO1P3 also seems to affect EMT as it regulates another EMT biomarker e-cadherin (21). Finally, lncRNA AGAP2-AS1 also affects EMT (23). This is one of the top lncRNAs with possible role in TMZ resistance, based on its elevated levels in resistant cells, as observed in this study. AGAP2-AS1 regulates EMT genes that can explain the observed effects of its downregulation on cellular behaviors.

Elucidation of a miRNA, downstream of HOTTIP in glioma cells, particularly those resistant to therapy, was another important goal of this study and for this we screened thirty potential miRNAs, based on available literature. Of these, we presented here the data we obtained on the top eight. One of the criteria for screening was proven targeting of miRNA in question by HOTTIP in addition to screening of some promising miRNAs based on their relevance to therapy resistance. miR-125b belongs to the category of miRNAs that have earlier been shown to be regulated by HOTTIP (31). Other miRNAs shown here that were reported to be regulated by HOTTIP in earlier studies are miR-216a (32), miR-615 (33), miR-148a (34) and miR-744 (35). All these five miRNAs that have earlier been reported to be regulated by HOTTIP are inversely associated with HOTTIP expression i.e. their expression is negatively regulated by HOTTIP, similar to the general reports in sponging of miRNAs by lncRNAs. However, for over study we also evaluated a few miRNAs that are positively correlated with therapy resistance. These are miR-10b, miR-21 and miR-221. There is a lot of published literature on involvement of these miRNAs in therapy resistance in different cancers (36–38), however, we are the first to provide a mechanism of miR-10b mediated EMT in the HOTTIP-regulated TMZ resistance of glioma cells.

miR-10b is a well-studied miRNA in terms for its role in therapy resistance. A report published a decade back suggested the role of miR-10b in conferring resistance against 5-fluorouracil in colorectal cancer cells (39). In an agreement with our findings, Zhang and co-workers found that miR-10b regulates EMT to participate in resistance against therapy (40). Their focus was on nasopharyngeal cancer and their study found a role of miR-10b in cisplatin resistance of nasopharyngeal carcinoma cells (40). In other reports, miR-10b was shown to regulate tamoxifen resistance in breast cancer cells (41) and cisplatin resistance of ovarian cancer cells (36).

Based on the results that we presented in this study, it is reasonable to conclude that lncRNA HOTTIP confers resistance against TMZ in glioma cells. Further, it induces miR-10b and EMT which finally converge in determining TMZ resistance. This work has a lot of clinical potential but first *in vivo* and clinical studies need to further verify these findings.

## Data Availability Statement

The original contributions presented in the study are included in the article/supplementary material. Further inquiries can be directed to the corresponding author.

## Author Contributions

ZLi and ML performed experiments. ZLi, ML, and PX analyzed results and performed statistical evaluations. ZLi and ZLu drafted manuscript. ZLu provided support and guidance. All authors proofread the manuscript. All authors contributed to the article and approved the submitted version.

## Conflict of Interest

The authors declare that the research was conducted in the absence of any commercial or financial relationships that could be construed as a potential conflict of interest.

## Publisher’s Note

All claims expressed in this article are solely those of the authors and do not necessarily represent those of their affiliated organizations, or those of the publisher, the editors and the reviewers. Any product that may be evaluated in this article, or claim that may be made by its manufacturer, is not guaranteed or endorsed by the publisher.
